# A Randomized, Double-Blind, Crossover Pilot Trial of Rice Endosperm Protein Supplementation in Maintenance Hemodialysis Patients

**DOI:** 10.1038/s41598-017-18340-8

**Published:** 2017-12-21

**Authors:** Michihiro Hosojima, Hisaki Shimada, Yoshitsugu Obi, Shoji Kuwahara, Ryohei Kaseda, Hideyuki Kabasawa, Hazuki Kondo, Mikio Fujii, Reiko Watanabe, Yoshiki Suzuki, Motoni Kadowaki, Shigeru Miyazaki, Akihiko Saito

**Affiliations:** 1 Department of Clinical Nutrition Science, Kidney Research Center, Niigata University Graduate School of Medical and Dental Sciences, Niigata, 951-8510 Japan; 2 Kidney Center, Shinrakuen Hospital, Niigata, 950-2087 Japan; 3 Harold Simmons Center for Kidney Disease Research & Epidemiology, Division of Nephrology & Hypertension, University of California, Irvine, CA 92868 USA; 4 Department of Nephrology, Osaka Graduate School of Medicine, Osaka, 565-0871 Japan; 5 Department of Applied Molecular Medicine, Kidney Research Center, Niigata University Graduate School of Medical and Dental Sciences, Niigata, 951-8510 Japan; 6 Division of Clinical Nephrology and Rheumatology, Kidney Research Center, Niigata University Graduate School of Medical and Dental Sciences, Niigata, 951-8510 Japan; 7 Rice Research Center, Kameda Seika Co. Ltd., Niigata, 950-0198 Japan; 8 Department of Health and Nutrition, University of Niigata Prefecture, Niigata, 950-8680 Japan; 9 Health Administration Center, Niigata University, Niigata, 950-2181 Japan; 10 Graduate School of Science and Technology, Niigata University, Niigata, 950-2181 Japan

## Abstract

In maintenance hemodialysis (MHD) patients, low protein intake is associated with protein-energy wasting, a risk factor that affects outcome. However, increased protein intake may lead to hyperphosphatemia and hyperkalemia, which are also mortality risk factors. Here, we evaluated the safety and effects of purified rice endosperm protein (REP), which contains less phosphorus and potassium than soy and casein proteins, as a supplemental protein source for MHD patients. This randomized, double-blind, placebo-controlled, crossover pilot study of REP supplementation (5 g/day × 4 weeks) was carried out in 50 Japanese adult MHD patients (1 dropped out); the primary outcome was the change in the urea kinetic-based normalized protein catabolic rate (nPCR), an indicator of protein intake in MHD patients. Intention-to-treat analyses of 24 patients in the REP-first group and 25 in the placebo-first group showed that REP supplementation increased nPCR significantly by 0.07 g/kg/day (95% confidence interval, 0.03–0.11), whereas changes in serum phosphorus and potassium concentrations were not different from the placebo. REP supplementation did not show a significant effect on other nutritional or metabolic parameters and no specific complications. In conclusion, purified REP with efficient bioavailability may be safe and useful for dietary supplementation in MHD patients.

## Introduction

Protein-energy wasting, a state of metabolic and nutritional derangement, is an important risk factor that affects the clinical outcome of patients with chronic kidney disease (CKD), particularly in those with end-stage renal disease on maintenance dialysis treatment^1,2^. While the recommended dietary protein intake is 0.6–0.8 g/kg ideal body weight/day in pre-dialysis patients with stage 3–5 CKD, the minimum protein requirement for end-stage renal disease patients on maintenance dialysis is suggested to be 1.2 g/kg ideal body weight/day due to additional protein catabolic stimuli including the inflammatory stimulus associated with the dialysis procedure and the loss of amino acids and albumin into the dialysate^1^. Thus, it should be noted that the low-protein diet recommended for pre-dialysis patients is not applicable to dialysis patients. Nevertheless, the normalized protein catabolic rate (nPCR), which is an index of dietary protein intake in this population, was reported to be suboptimal across countries, with a median value ranging from 0.91 g/kg/day in Japan to 1.10 g/kg/day in Spain^3^. This suggests the need for protein supplementation for the majority of maintenance hemodialysis (MHD) patients.

However, increased protein intake may lead to hyperphosphatemia and hyperkalemia, which are risk factors for mortality^4,5^. In addition, high acidogenic food intake, high salt and fluid intake, and lower vegetable intake may be other risk factors associated with increased protein intake^6^. A previous study reported a U-shaped association between nPCR and mortality in MHD patients; where the best survival was observed between 1.0 and 1.4 g/kg/day, and both lower (<0.8 g/kg/day) and higher (>1.4 g/kg/day) nPCR were associated with greater mortality^7^. Another study also suggested greater survival in MHD patients whose serum phosphorus decreased but whose nPCR increased, when compared with those whose serum phosphorus and nPCR rose over 6 months^8^. Therefore, it is critical to establish dietary protein sources at least containing less phosphorus and potassium to prevent protein-energy wasting by enhancing protein intake in MHD patients.

Rice, one of the most important cereals in the world, is a major plant source of both energy and protein^9,10^. Recently, a simple, mass preparative procedure for rice endosperm protein (REP) was developed using an alkali extraction method^11^. This isolate is characterized by >80% crude protein, with lower amounts of phosphorus and potassium compared with soy^12^ and casein proteins^13^ (Table 1). The digestibility of native REP in humans is 88%^14^, with the major indigestible component being prolamin. The alkali extraction procedure changes the digestibility of prolamin markedly^15^ and improves the bioavailability of whole REP^11,16^.

**Table 1:** Composition of the REP powder used in this study and of soy and casein proteins as reference.

	REP*	Soy protein**	Casein protein***
Water (%)	2.5 ± 0.3	4.98	10.8
Protein (%)	91.2 ± 1.0	88.32	86.1
Phosphorus (mg/100 g)	95 ± 16	776	730
Potassium (mg/100 g)	<1	81	2.7
Sodium (mg/100 g)	17.3 ± 2.7	1005	2.7
Calcium (mg/100 g)	31.4 ± 5.3	178	24.2
Magnesium (mg/100 g)	5.1 ± 0.7	39	2.6
Cadmium (ppm)	0.14 ± 0.17	N/A	N/A

^*^Average of 5 batches ± standard deviation. **From ref.^12^. ***From ref.^13^. REP, rice endosperm protein; N/A, not available.

In this clinical trial, we investigated the effects of REP supplementation on nPCR, mineral metabolism, and nutritional and metabolic parameters in MHD patients with insufficient protein intake and inadequate nutritional status.

## Results

### Participant Characteristics

**Figure 1:**
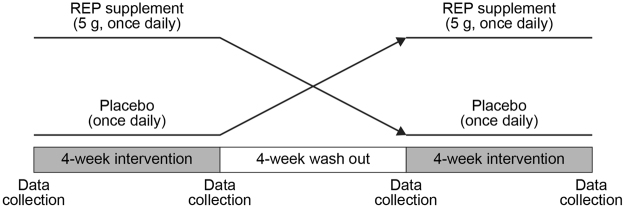
Overall crossover design of this study. Data collection: anthropometric measurements and blood drawing. REP, rice endosperm protein.

**Figure 2:**
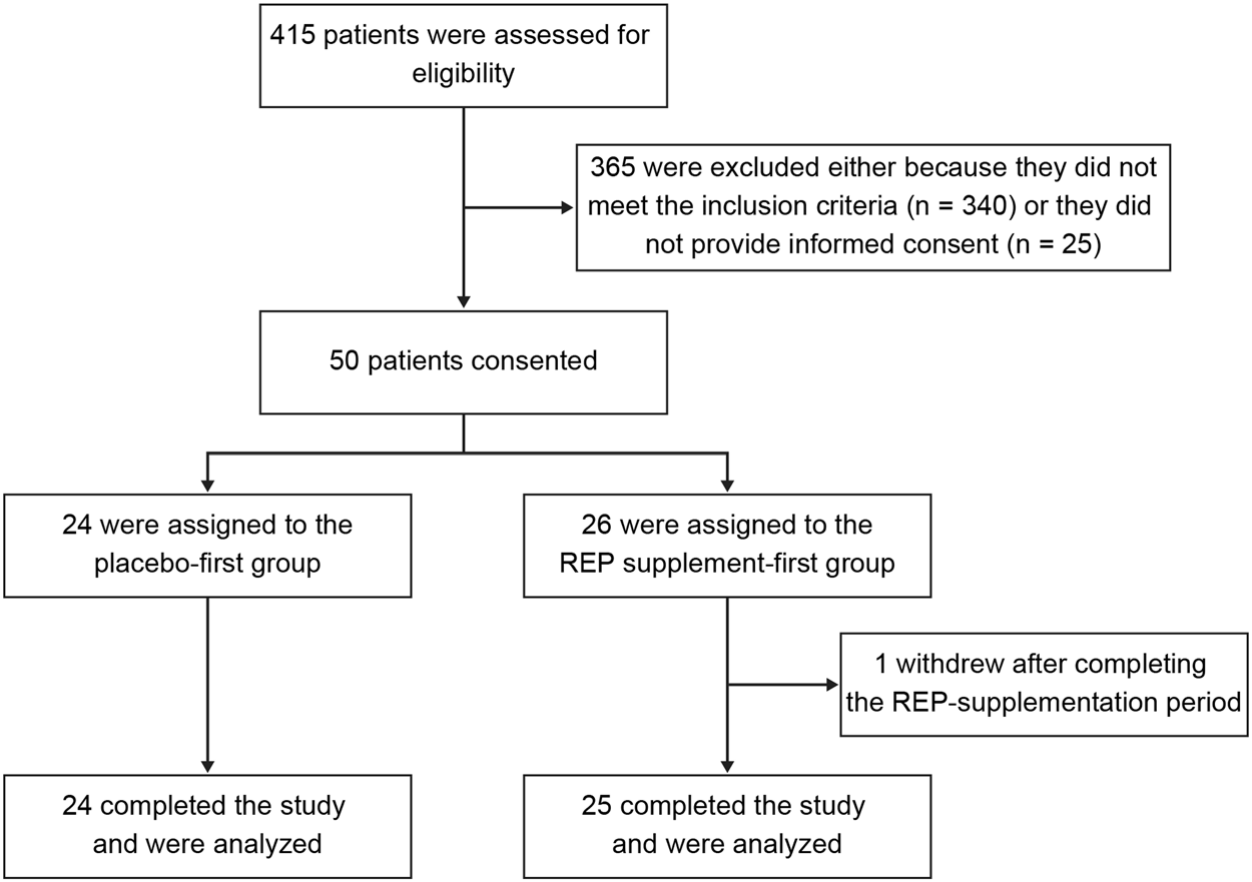
Enrollment, randomization, and follow-up of study participants. REP, rice endosperm protein.

The outline of this single-center, double-blind, placebo-controlled crossover pilot trial is shown in Fig. 1. We screened 415 MHD patients and enrolled 50 participants satisfying the inclusion criteria who were allocated randomly to receive REP first or placebo first (Fig. 2). The composition of the REP powder and the constituents of the intervention foods are shown in Tables 1 and 2, respectively. One participant dropped out of the REP-first group after completing the REP supplementation period because of hospitalization for a skin ulcer on the right arm, leaving 25 in the REP-first group and 24 in the placebo-first group for analysis (Fig. 2). Table 3 shows the clinical characteristics of the 49 participants at baseline. The mean age was 68 ± 10 years, 41% were female, mean serum albumin was 3.3 ± 0.3 g/dL, mean body mass index was 20.6 ± 1.3 kg/m^2^, and mean nPCR was 0.88 ± 0.16 g/kg/day. The participants had been on MHD for a median duration of 10 years (interquartile range, 4–17 years; range, 2–44 years). They were all anuric, so their residual kidney function was minimal or negligible. Phosphate binders, vitamin D receptor activators, cinacalcet, and potassium binders had been prescribed to 40, 27, 7, and 17 participants, respectively, and the medications were constant from 1 month before and until the end of the study. Sensitivity analyses, carried out by excluding 6 patients with adverse events (5 infection episodes and 1 gastrointestinal bleeding), which were diagnosed not to be due to the intake of the intervention foods, resulted in consistent results (data not shown). Adherence to REP and placebo in the study patients was both 99.5%.

**Table 2:** Constituents of the intervention foods.

Materials	Content (g)	
	REP-containing	Placebo
REP powder	5.6*	—
Polydextrose	4	4
Erythritol	3.5	3.5
Agar	0.25	0.25
Gelling agents	0.025	0.025
Hydroxypropylated distarch phosphate	1	1
Sweetener (sucralose, acesulfame K)	0.005	0.005
Sweetener (sucralose)	0.005	0.005
Sodium ascorbate	0.05	0.05
Flavors	0.1	0.1
Food coloring	0.1	0.1
Water	Filled up to 50 g	Filled up to 50 g
Energy (kcal)	31.6	7.7

^*^Quantity of the REP powder was adjusted based on its protein content to achieve a 5 g dose of REP. REP, rice endosperm protein.

**Table 3:** Baseline characteristics of the participants.

	**Placebo-first (n = 24)**	**REP-first (n = 25)**
Age (years)	69 ± 11	66 ± 9
Male (%)	58%	58%
*Comorbidities*		
Diabetes (%)	29.2%	32.0%
*Anthropometrics*		
Dry weight (kg)	52.4 ± 6.2	52.8 ± 6.5
Skeletal muscle (kg)*	20.1 ± 3.4	20.8 ± 4.5
Fat mass (kg)*	14.1 ± 4.4	13.5 ± 4.2
Lean body mass (kg)*	38.0 ± 5.9	39.1 ± 7.5
MAC (cm)	23.8 ± 1.5	24.0 ± 2.3
Skinfold thickness (cm)	9.1 ± 4.2	8.0 ± 4.0
Single-pool Kt/V	1.19 ± 0.17	1.26 ± 0.16
nPCR (g/kg/day)	0.84 ± 0.16	0.91 ± 0.16
*Laboratory measurements*		
Total protein (g/dL)	6.05 ± 0.33	6.22 ± 0.44
Albumin (g/dL)	3.20 ± 0.31	3.33 ± 0.23
Prealbumin (g/dL)	23.4 ± 6.6	26.2 ± 6.6
RBP (mg/dL)	7.2 ± 1.7	8.4 ± 1.9
Transferrin (mg/dL)	206 ± 42	218 ± 47
Total cholesterol (mg/dL)	141 ± 27	149 ± 31
Potassium (mEq/L)	4.7 ± 0.8	5.2 ± 0.8
Corrected calcium (mg/dL)	9.4 ± 0.5	9.4 ± 0.5
Phosphate (mg/dL)	4.9 ± 1.2	5.5 ± 1.2
25(OH)D (ng/mL)	19.5 ± 8.4	20.1 ± 7.3
1,25(OH)_2_D (pg/mL)	13.7 (IQR, 7.1–19.4)	11.5 (IQR, 8.2–18.9)
Intact PTH (pg/mL)	117 (IQR, 55–270)	122 (IQR, 73–198)
1–84 PTH (pg/mL)	53 (IQR, 24–127)	63 (IQR, 34–89)
Intact FGF23 (pg/mL)	1,475 (IQR, 533–6,565)	4,605 (IQR, 1,320–13,500)
Hemoglobin (g/dL)	9.9 ± 1.5	10.1 ± 1.5
Glycated hemoglobin (%)	5.4 (IQR, 4.9–6.6)	5.3 (IQR, 4.7–6.1)
C-reactive protein (mg/dL)	0.07 (IQR, 0.04–0.21)	0.08 (IQR, 0.04–0.22)

Data are expressed as mean (standard deviation) or median (IQR) according to their distribution. *Missing in 3 patients. 1,25(OH)_2_D, 1,25-dihydroxyvitamin D; FGF23, fibroblast growth factor-23; 25(OH)D, 25-hydroxyvitamin D; IQR, interquartile range; MAC, mid-upper arm circumference; nPCR, normalized protein catabolic rate; PTH, parathyroid hormone; RBP, retinol-binding protein; REP, rice endosperm protein.

### Primary Outcome

**Figure 3:**
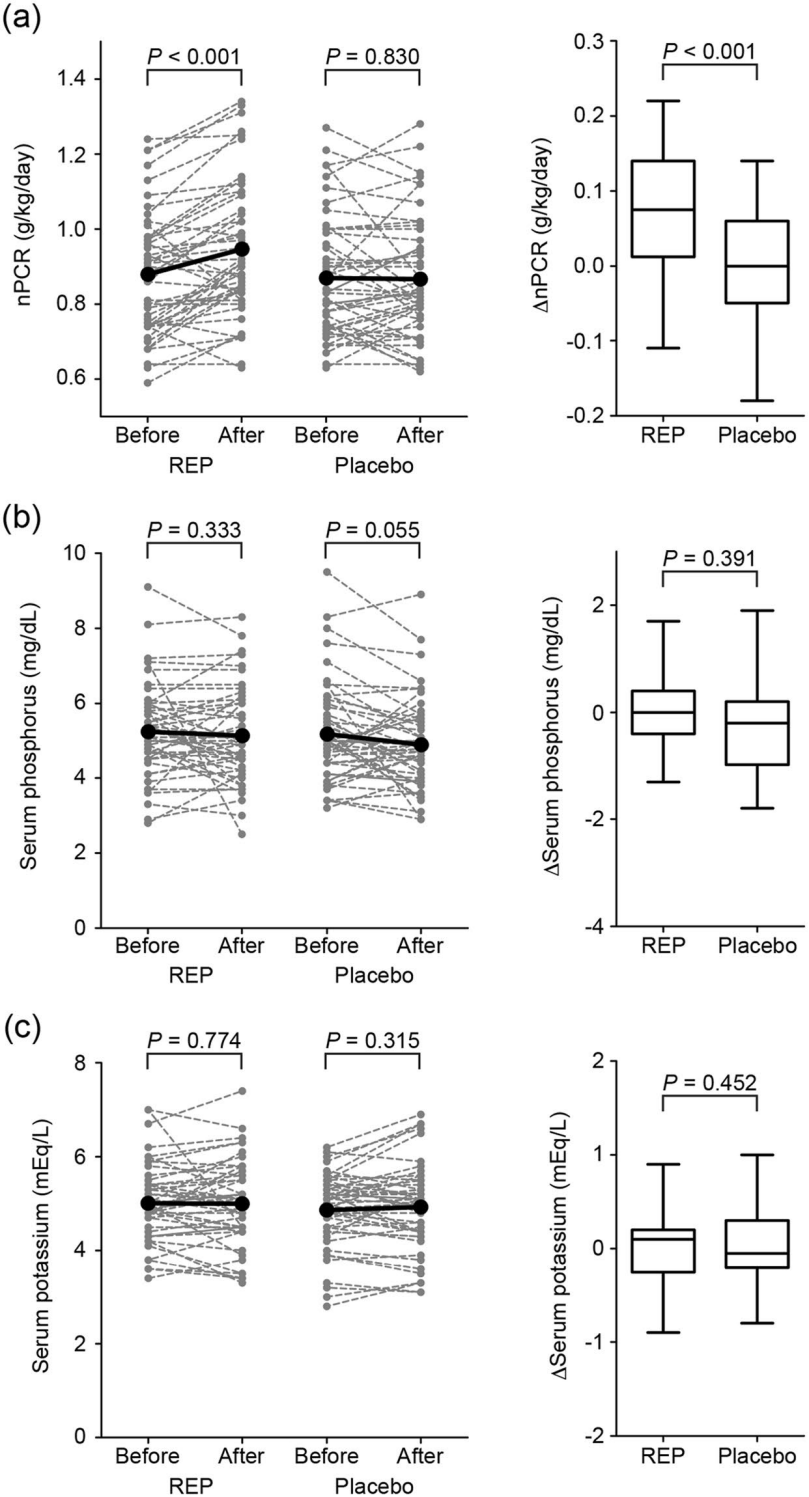
Analyses of the normalized protein catabolic rate (nPCR) and serum phosphorus and potassium concentrations. The within-group changes from the beginning (Before) to the end (After) of rice endosperm protein (REP) or placebo intervention period (left panels) and their between-group differences (right panels) in (**a**) normalized protein catabolic rate (nPCR), (**b**) serum phosphorus, and (**c**) potassium concentrations. Left panels: dotted lines represent changes in the individual measurements and thick lines represent changes in the mean values. Right panels: box-and-whisker plots. The ends of the box represent the upper and lower quartiles. The horizontal line in the box marks the median. The whiskers are extended to the highest and lowest values.

The primary outcome of this study was the change in nPCR. In intention-to-treat analyses, REP significantly increased nPCR from 0.88 ± 0.16 to 0.95 ± 0.18 g/kg/day, by 0.07 g/kg/day (95% confidence interval [CI], 0.03–0.11), compared with placebo (*P* < 0.001) (Fig. 3).

### Secondary Outcomes

Serum minerals, nutritional and metabolic parameters, and adverse effects were also evaluated in this study. Changes in serum phosphorus and potassium concentrations were not significantly different between the groups (0.18 [95% CI, −0.23 to 0.58] mg/dL and −0.08 [95% CI, −0.27 to 0.12] mg/dL, respectively) (*P* = 0.391 and 0.452, respectively) (Fig. 3). Table 4 shows the “within-group” changes in other parameters from baseline to the end of each intervention period and their “between-group” differences. Of note, phosphate metabolism-related parameters (i.e., intact parathyroid hormone [PTH], 1–84 PTH, and intact fibroblast growth factor-23 [FGF23]) did not change in the REP group. Single-pool Kt/V, an index of dialysis dose, was not significantly different between the groups. There were also no significant between-group differences in relation to changes in anthropometric data (e.g., dry weight, skeletal muscle, fat mass, lean body mass, mid-arm circumference, and skinfold thickness) or changes in markers of nutritional status (e.g., serum total protein, albumin, prealbumin, retinol-binding protein [RBP], and transferrin). There were no differences in the other parameters of blood analysis between the groups. During the intervention and follow-up periods, there were no specific complications associated with REP or placebo supplementation.

**Table 4:** Within-group changes from baseline to the end of each intervention period and their between-group differences.

	N	Intervention	Within-group changes	Between-group differences in within-group changes	
			Mean (%CI)	Mean (%CI)	*P*-value
Dry weight (kg)	49	REP	0.0 (−0.1 to 0.0)	0.0 (−0.1 to 0.1)	0.877
		Placebo	−0.1 (−0.1 to 0.1)		
Skeletal muscle (kg)	39	REP	−0.1 (−0.2 to 0.1)	−0.2 (−0.6 to 0.1)	0.182
		Placebo	0.2 (−0.1 to 0.5)		
Fat mass (kg)	39	REP	0.1 (−0.2 to 0.3)	0.0 (−0.4 to 0.4)	0.980
		Placebo	0.1 (−0.2 to 0.4)		
Lean body mass (kg)	39	REP	−0.1 (−0.4 to 0.2)	−0.2 (−0.6 to 0.3)	0.450
		Placebo	0.1 (−0.3 to 0.3)		
MAC (cm)	48	REP	0.3 (0.1 to 0.5)	0.0 (−0.4 to 0.4)	0.967
		Placebo	0.3 (0.0 to 0.7)		
Skinfold thickness (cm)	48	REP	0.5 (0.0 to 1.0)	0.0 (−1.0 to 0.9)	0.929
		Placebo	0.5 (−0.2 to 1.3)		
Single-pool Kt/V	49	REP	−0.01 (−0.04 to 0.02)	0.0 (−0.1 to 0.0)	0.199
		Placebo	0.02 (−0.01 to 0.05)		
Total protein (g/dL)	49	REP	−0.04 (−0.12 to 0.04)	−0.04 (−0.17 to 0.09)	0.518
		Placebo	0.00 (−0.08 to 0.08)		
Albumin (g/dL)	49	REP	−0.02 (−0.09 to 0.04)	−0.08 (−0.17 to 0.01)	0.075
		Placebo	0.06 (0.01 to 0.11)		
Prealbumin (g/dL)	49	REP	−0.8 (−1.7 to 0.1)	−0.5 (−1.8 to 0.8)	0.439
		Placebo	−0.3 (−1.1 to 0.4)		
RBP (mg/dL)	49	REP	−0.1 (−0.3 to 0.2)	−0.1 (−0.5 to 0.3)	0.474
		Placebo	0.1 (−0.1 to 0.3)		
Transferrin (mg/dL)	49	REP	−3.35 (−9.79 to 3.09)	−2.9 (−11.8 to 6.1)	0.531
		Placebo	−1.59 (−8.02 to 4.83)		
Total cholesterol (mg/dL)	49	REP	0 (−4 to 3)	−4.1 (−9.1 to 0.9)	0.103
		Placebo	4 (1 to 7)		
Corrected calcium (mg/dL)	49	REP	0.04 (−0.04 to 0.13)	0.01 (−0.14 to 0.16)	0.888
		Placebo	0.03 (−0.05 to 0.12)		
25(OH)D (ng/mL)	49	REP	−0.8 (−2.3 to 0.8)	0.4 (−1.8 to 2.5)	0.741
		Placebo	−1.1 (−2.7 to 0.6)		
Ln(1,25D [pg/mL])	49	REP	−0.04 (−0.12 to 0.04)	0.00 (−0.10 to 0.11)	0.953
		Placebo	−0.04 (−0.11 to 0.03)		
Ln(intact PTH [pg/mL])	49	REP	−0.07 (−0.17 to 0.03)	−0.06 (−0.21 to 0.09)	0.425
		Placebo	−0.01 (−0.10 to 0.08)		
Ln(1–84 PTH [pg/mL])	49	REP	0.08 (−0.19 to 0.03)	−0.08 (−0.23 to 0.06)	0.257
		Placebo	0.00 (−0.09 to 0.10)		
Ln(FGF23 [pg/mL])	49	REP	0.03 (−0.12 to 0.19)	0.15 (−0.07 to 0.36)	0.172
		Placebo	−0.11 (−0.27 to 0.05)		
Hemoglobin (g/dL)	49	REP	0.1 (−0.2 to 0.3)	0.0 (−0.3 to 0.3)	0.915
		Placebo	0.0 (−0.1 to 0.2)		
Ln(glycated hemoglobin [%])	49	REP	0.0 (0.0 to 0.0)	0.0 (0.0 to 0.1)	0.189
		Placebo	0.0 (0.0 to 0.0)		
Ln(C-reactive protein [mg/dL])	49	REP	0.00 (−0.29 to 0.29)	0.04 (−0.32 to 0.40)	0.814
		Placebo	−0.04 (−0.27 to 0.18)		

1,25(OH)_2_D, 1,25-dihydroxyvitamin D; CI, confidence interval; FGF23, fibroblast growth factor-23; 25(OH)D, 25-hydroxyvitamin D; MAC, mid-upper arm circumference; PTH, parathyroid hormone; RBP, retinol-binding protein; REP, rice endosperm protein.

### Self-Administered Diet History Questionnaire Analysis

The composition of nutrients analyzed from the self-administered diet history questionnaire (DHQ) at the beginning and end of this study is shown in Table 5. There were no significant differences in the intake of energy, carbohydrate, fat, protein, phosphorous, or potassium between before and after the study.

**Table 5:** Energy and nutrient intakes at the beginning and end of this study according to diet history questionnaire analysis.

Dietary intake	Pre	Post	*P*-value
Energy (kcal/day)	1,491 ± 477	1,540 ± 516	0.348
Carbohydrate (%energy)	60.7 ± 7.7	59.8 ± 7.9	0.374
Fat (%energy)	21.6 ± 5.8	21.9 ± 6.0	0.670
Protein (%energy)	12.6 ± 2.3	13.2 ± 2.1	0.075
Phosphorous (mg/1,000 kcal)	963 ± 263	1,006 ± 218	0.393
Potassium (mg/1,000 kcal)	453 ± 86	462 ± 81	0.179

## Discussion

In this randomized, double-blind, crossover pilot trial, oral REP supplementation (5 g/day) for 4 weeks significantly increased nPCR without significant changes in nutrient intake and serum concentrations of phosphorus and potassium in MHD patients with insufficient protein intake. There were no specific complications associated with REP or placebo supplementation. In addition, there were no significant changes in anthropometric data or markers of nutritional status by REP supplementation, probably due to the relatively short-term study period.

Values of nPCR may be underestimated among MHD patients with residual kidney function^17^. However, most of our participants were long-term MHD patients with a median duration of 10 years (interquartile range, 4–17 years; range, 2–44 years) and they were all anuric. Therefore, they were likely to have little or no residual kidney function. Additionally, the primary outcome of this study was the change in nPCR, and hence, the influence of residual kidney function is considered to be minimal given the nature of this randomized cross-over trial design with a relatively short-term study period of 4 weeks.

Some randomized controlled studies of protein supplementation in MHD patients have been reported. Moretti *et al*. reported that liquid hydrolyzed collagen protein supplement given to patients treated with MHD or peritoneal dialysis (45 g for MHD and 105 g for peritoneal dialysis patients per week) for 6 months significantly increased nPCR and serum albumin concentration^18^. Sezer *et al*. found that 2–3 daily servings of supplement containing 14 g cow milk-derived proteins, such as casein, for 6 months tended to increase nPCR and significantly improved serum albumin levels and anthropometric measures in malnourished MHD patients^19^. However, these studies did not report changes in serum phosphorus or potassium concentration. Casein protein supplementation (6.6 g/day) in MHD patients for 4 months improved prealbumin levels, but increased serum phosphorus levels^20^. Meanwhile, a 6-week single-arm pilot study involving 13 MHD patients was conducted with 225 g pasteurized liquid egg white as a supplemental protein source^21^, which contains biologically high-value protein but relatively low phosphorus and cholesterol^22,23^. The study showed a decrease in serum phosphorus and an increase in serum albumin, requiring further controlled studies. In addition, it remains to be tested whether increasing nPCR by protein supplementation would decrease the mortality of MHD patients.

The source of dietary protein (animal or plant) is an important nutritional issue. Excess animal protein intake was found to be associated with an increased risk of diabetes in a European prospective cohort study with 38,094 participants^24^. A diet with a higher proportion of protein from plant sources was associated with improved metabolic acidosis and blood pressure reduction^25^ as well as lower all-cause mortality in patients with CKD^26^. Plant-sourced protein is also known to have beneficial effects on phosphorus metabolism in CKD patients^27,28^, although there may be other unknown favorable factors involved. However, as shown in Table 1, purified soy protein was reported to contain relatively high levels of phosphorus (776 mg/100 g) and potassium (81 mg/100 g)^12^. In contrast, the potassium content of our REP preparation is negligible, which is another beneficial factor in addition to its low phosphorus content for its use as a supplement for MHD patients.

Protein supplementation may not be solely sufficient to improve protein-energy wasting in MHD patients. Indeed, protein supplementation with ricotta cheese alone did not increase muscle mass in sarcopenic elderly men^29^. Kim *et al*. suggested that exercise and amino acid supplementation together may be effective in enhancing not only muscle strength but also the combined variables of muscle mass and walking speed and of muscle mass and strength in sarcopenic women^30^. In MHD patients, Majchrzak *et al*. reported that resistance exercise augments the protein anabolic effects of oral nutritional supplementation^31^.

The limitations of this pilot study are the shortness of the trial period and the small number of participants. In addition, this was a single-center study. The clinical relevance of an increase in nPCR also remains to be investigated. The placebo intervention food was prepared with the same recipe and packaging as the test food, but without REP. However, the taste of both types of intervention food—REP-containing jelly and placebo jelly—may not have been completely the same, although it would have been difficult for the participants to distinguish one from the other.

In conclusion, as it has low phosphorus and potassium content as well as efficient bioavailability, REP may be a safe and useful source of protein supplementation for MHD patients. In the future, long-term studies with adequate sample size, as well as with combined exercise intervention, are needed to evaluate whether REP supplementation improves the clinical outcome of MHD patients.

## Methods

### Participants and Study Design

This was a single-center, double-blind, placebo-controlled crossover pilot trial, with change in the nPCR as the primary outcome. Serum minerals, nutritional and metabolic parameters, and adverse effects were also evaluated. The study participants were enrolled from July 29 to August 31, 2013, and were followed up for 12 weeks by M.H., H.S., and S.M. in Shinrakuen Hospital, Niigata, Japan. The inclusion criteria were: (1) age ≥ 20 years, (2) on maintenance HD ≥ 2 years, (3) serum albumin < 3.8 g/dL, (4) body mass index ≥ 19 and <23 kg/m^2^, (5) change in dry weight during the last 6 months < 5%, (6) nPCR < 1.2 g/kg/day, and (7) ability to provide informed consent and come for all visits. The exclusion criteria were: (1) severe heart disease (New York Heart Association III or IV), (2) severe hepatic insufficiency, (3) apparent signs of current systemic infection or sepsis requiring the active use of intravenous antibiotics, (4) perioperative status, (4) severe traumatic injury, and (5) any food allergy. The study participants were all anuric, that is, their urine volume was less than 100 mL/day, ascertained by questionnaires at baseline. A random allocation table was generated separately by computer at the central study facility, using a block size of 4 with stratification by gender. Y.O. allocated the participants in a 1:1 ratio to receive initially either placebo or 5 g REP-containing jelly supplement to eat once daily for 4 weeks. This amount was determined by the water intake restriction for MHD patients and a previous preliminary safety evaluation. The assigned allocation was concealed from the participants and care providers throughout the study period. After 4 weeks of a given intervention and after a wash-out period of 4 weeks, the participants received the other intervention. The participants continued their usual diet and physical activities during the study period. Pre- and post-HD blood samples were obtained at the beginning and end of each intervention period (Fig. 1). This study was approved by the institutional review boards of Niigata University and Shinrakuen Hospital in accordance with the principles embodied in the Declaration of Helsinki, and all participants gave written informed consent. The study was registered with the University Hospital Medical Information Network–Clinical Trials Registry (UMIN000010876; June 10, 2013).

### Preparation of REP

REP was extracted from rice flour of regular Japonica rice *Koshihikari* using the alkaline extraction method^11^ with modifications. Rice flour (100 kg) was mixed with a 0.2% NaOH solution (400 L, approximately pH 12.5) and extracted overnight at room temperature. The mixture was then centrifuged to collect the supernatant. The supernatant was heated at 50 °C and HCl was added to adjust the pH to 7.0 ± 0.2. REP aggregate was developed by heating the supernatant at 80 °C for 15 min. To remove minerals and heavy metals such as potassium and cadmium, REP aggregate was washed 4 times with a diluted HCl solution after adjusting the pH to 4.0 ± 0.2. Washed REP was neutralized to pH 7.0 ± 0.2 and pasteurized by heating at 80 °C for 15 min. Finally, REP aggregate was dehydrated and lyophilized. Five batches of REP powder were prepared. The composition of REP powder is shown in Table 1. The concentrations of the following were then measured: protein, using the Kjeldahl method; phosphorus, with ammonium vanadomolybdate absorption photometry; potassium, sodium, and cadmium, with atomic absorption spectrophotometry; and calcium and magnesium, with inductively coupled plasma-atomic emission spectroscopy. For reference, the compositions of soy^12^ and casein proteins^13^ are also shown in Table 1. The amino acid compositions of REP and of soy and casein proteins are shown separately in Supplementary Table 1.

### Preparation of the Intervention Food

As MHD patients generally restrict their water intake, the intervention food was prepared with a limited quantity of water to produce a thick jelly. Two types of intervention food were prepared—REP-containing jelly and placebo jelly—by Kameda Seika Co., Ltd. (Niigata, Japan). The composition of each intervention food is shown in Table 2. Polydextrose (Litesse Ultra®), erythritol (Zerose®), hydroxypropyl distarch phosphate (Farinex LCF®), and sodium ascorbate were purchased from Danisco A/S (Copenhagen, Denmark), Cargill, Inc. (Minnetonka, MN, USA), AVEBE U.A. (Veendam, the Netherlands), and Fuso Chemical Co., Ltd. (Osaka, Japan), respectively. Agar (Gelup J-1630®), gelling agent (Kelcogel LT-100®), sweetener (Sansweet SA-8020® and Sansweet SU-100®), flavors (Azuki flavor 68901® and Marron oil No. 43471®), and food coloring (San brown AC® and Carotene base 80-SV®) were purchased from San-Ei Gen F.F.I. Co., Ltd. (Osaka, Japan). To maintain the acceptability of the intervention food, two types were made with different flavors and food colorings—Azuki bean-type (with Azuki flavor 68901® and San brown AC®) and chestnut-type (with Marron oil No.43471® and Carotene base 80-SV®)—and were distributed alternatively every other week. All materials were mixed with REP and boiled. Then, the jelly mixture was packaged into film-covered opaque cups (volume, 50 mL) without any indication to ensure double-blindness. Placebo food was prepared with the same recipe but without REP. Finally, the jellies were sterilized using a retort machine. Intervention foods used for 2 weeks were prepared every other week and were provided to each participant based on the random allocation within 4 days of sterilization.

### Laboratory Methods

Pre-HD venous blood samples were obtained at the beginning of the week. Routine biochemical parameters and RBP, transferrin, 25(OH)D, 1,25(OH)_2_D, 1–84 PTH, and intact FGF23 were analyzed. Serum phosphate and albumin were measured by the molybdate direct method and modified bromocresol purple method, respectively. RBP and transferrin levels were measured by latex agglutination turbidimetry and turbidimetric immunoassay (Nittobo Medical Co., Ltd., Tokyo, Japan), respectively. Double-antibody radioimmunoassays were used to measure the levels of 25(OH)D (DiaSorin, Inc., Stillwater, MI, USA) and 1,25(OH)_2_D (Immunodiagnostic Systems Ltd., Boldon, UK). Levels of 1–84 PTH were measured by a chemiluminescent enzyme immunoassay (Fujirebio, Inc., Tokyo, Japan) and those of intact FGF23 were measured using an enzyme-linked immunosorbent assay (Kainos Laboratories, Inc., Tokyo, Japan). nPCR and Kt/V were calculated using a formal single-pool model of urea kinetics^32^.

Body composition was assessed by segmental multiple frequency bioelectric impedance measurements at baseline and at the end of each intervention period using tetra polar 8-point tactile electrodes (InBody S20; BioSpace, Seoul, South Korea).

Dietary habits were assessed by a validated DHQ at the beginning and end of the study. Details of the structure, calculation of dietary intake, and validity of the DHQ for commonly studied macronutrient intake are available elsewhere^33^.

### Statistical Analysis

This was a pilot study without sample size calculation. The intention-to-treat principle was employed for primary analyses, and a two-sided *P*-value of <0.05 was considered significant. Summary data of all variables are expressed as mean ± standard deviation and were compared using a paired t-test or signed-rank test according to their distribution. For 25(OH)D (*P = *0.002), FGF23 (*P = *0.001), and transferrin (*P = *0.019), we found significant differences in the effects of REP supplementation between the intervention orders, suggesting incomplete waning of the treatment effect during the washout period. Therefore, these variables were analyzed using linear mixed-effects models with participant-level random intercepts to test the effect of REP supplementation, adjusting for the period in which REP supplementation was administered. Sensitivity analyses were conducted after excluding patients who dropped out of this study due to severe adverse events. All analyses were conducted using Stata/IC 12.1 for Windows (StataCorp LP, College Station, TX, USA).

### Data Availability

The datasets generated during and/or analysed during the current study are available from the corresponding author on reasonable request.

## Electronic supplementary material

Supplementary Table 1
