# Supplementary material for: A Randomized, Double-Blind, Crossover Pilot Trial of Rice Endosperm Protein Supplementation in Maintenance Hemodialysis Patients
Source: Sci Rep. 2017 Dec 21;7:18003. doi: 10.1038/s41598-017-18340-8 (PMC5740176; doi:10.1038/s41598-017-18340-8)
Supplement: Supplementary file 1 — Supplementary Table 1 [file 41598_2017_18340_MOESM1_ESM.doc]

| Amino acids  (μmol/mg protein) | REP* | Soy protein** | Casein protein* |
| --- | --- | --- | --- |
| Aspartic acid + Asparagine | 0.73 | 0.87 | 0.55 |
| Threonine | 0.31 | 0.30 | 0.36 |
| Serine | 0.55 | 0.49 | 0.58 |
| Glutamic acid + Glutamine | 1.23 | 1.34 | 1.55 |
| Glycine | 0.62 | 0.54 | 0.25 |
| Alanine | 0.65 | 0.46 | 0.35 |
| Valine | 0.42 | 0.40 | 0.50 |
| Isoleucine | 0.29 | 0.37 | 0.36 |
| Leucine | 0.66 | 0.59 | 0.72 |
| Tyrosine | 0.23 | 0.20 | 0.22 |
| Phenylalanine | 0.34 | 0.31 | 0.32 |
| Lysine | 0.23 | 0.41 | 0.50 |
| Histidine | 0.15 | 0.17 | 0.17 |
| Arginine | 0.50 | 0.43 | 0.20 |
| Proline | 0.43 | 0.49 | 0.17 |

Supplementary Table 1. Amino acid composition of REP and of soy and casein proteins as reference. *From reference 13. **Modified from reference 12. REP, rice endosperm protein.
